# FusC, a member of the M16 protease family acquired by bacteria for iron piracy against plants

**DOI:** 10.1371/journal.pbio.2006026

**Published:** 2018-08-02

**Authors:** Rhys Grinter, Iain D. Hay, Jiangning Song, Jiawei Wang, Don Teng, Vijay Dhanesakaran, Jonathan J. Wilksch, Mark R. Davies, Dene Littler, Simone A. Beckham, Ian R. Henderson, Richard A. Strugnell, Gordon Dougan, Trevor Lithgow

**Affiliations:** 1 Infection and Immunity Program, Biomedicine Discovery Institute and Department of Microbiology, Monash University, Clayton, Australia; 2 Institute of Microbiology and Infection, School of Immunity and Infection, University of Birmingham, Birmingham, United Kingdom; 3 Infection and Immunity Program, Biomedicine Discovery Institute and Department of Biochemistry & Molecular Biology, Monash University, Clayton, Australia; 4 Department of Microbiology and Immunology, The Peter Doherty Institute, The University of Melbourne, Parkville, Australia; 5 Wellcome Trust Sanger Institute, Hinxton, Cambridge, United Kingdom; Brigham and Women’s Hospital, United States of America

## Abstract

Iron is essential for life. Accessing iron from the environment can be a limiting factor that determines success in a given environmental niche. For bacteria, access of chelated iron from the environment is often mediated by TonB-dependent transporters (TBDTs), which are β-barrel proteins that form sophisticated channels in the outer membrane. Reports of iron-bearing proteins being used as a source of iron indicate specific protein import reactions across the bacterial outer membrane. The molecular mechanism by which a folded protein can be imported in this way had remained mysterious, as did the evolutionary process that could lead to such a protein import pathway. How does the bacterium evolve the specificity factors that would be required to select and import a protein encoded on another organism’s genome? We describe here a model whereby the plant iron–bearing protein ferredoxin can be imported across the outer membrane of the plant pathogen *Pectobacterium* by means of a Brownian ratchet mechanism, thereby liberating iron into the bacterium to enable its growth in plant tissues. This import pathway is facilitated by FusC, a member of the same protein family as the mitochondrial processing peptidase (MPP). The Brownian ratchet depends on binding sites discovered in crystal structures of FusC that engage a linear segment of the plant protein ferredoxin. Sequence relationships suggest that the bacterial gene encoding FusC has previously unappreciated homologues in plants and that the protein import mechanism employed by the bacterium is an evolutionary echo of the protein import pathway in plant mitochondria and plastids.

## Author summary

Earth’s carbon cycle depends on saprophytic microbes to rot old or diseased plant matter and recycle carbon from that biomass. Some bacteria (phytopathogens) have evolved to cause disease and rot in even healthy plants and may have utility as biological control agents against noxious weeds. To understand the mechanisms driving each of these scenarios has significance in environmental engineering and agriculture. Access to iron is a limiting factor for bacteria-mediated plant rot. Here, we show how a plant-pathogenic bacteria has reevolved a mechanism, analogous to the protein import pathways that evolved in plant plastids and mitochondria, to import the plant iron–bearing protein ferredoxin from plant tissue. The study is based on structural and biophysical characterization of a key M16 family protease, FusC, resident inside the bacterial outer membrane.

## Introduction

TonB-dependent transporters (TBDTs) are 22-stranded β-barrel proteins integrated in the bacterial outer membrane. They contain a plug domain inserted inside the β-barrel that can be actively removed by the inner membrane protein TonB to provide access for the import of vitamins, chelated metals, and other cofactors essential to bacterial viability [1]. These various cofactors need to be small enough to pass through the internal channel of the TBDT, a channel whose internal diameter is approximately 20 Å [1–3]. TBDTs also serve as an outer membrane channel to initiate the import of bacteriocins, a class of protein antibiotics that are larger than the channel diameter but that have evolved to unfold in response to engagement with their specific target TBDT [4, 5]. The genes for bacteriocins have coevolved with the genes for the TBDTs, producing an exquisitely specific and deadly system of protein transport to control bacterial population growth [6].

In the case of bacterial pathogens, iron is acquired from host tissues, in a process referred to as iron piracy [7]. This process too is mediated by TBDTs. In some cases, the iron is captured by small siderophores, the genes for which coevolved with those of the TBDTs such that the size of the siderophore conveniently fits within the constraints imposed by the channel formed by the TBDT [1–3]. However, there are also several well-documented examples in which iron is acquired by pathogens through direct extraction from host proteins [7, 8]. Proteins such as ferredoxin, hemoglobin, and others are bigger than the TBDT channel. Nonetheless, under iron limitation, pathogens like *Pectobacterium* can remain viable and virulent using proteins such as ferredoxin, pirated from their plant host to supply iron [9]. How this important protein transport pathway is mediated by the bacterium had remained unknown and difficult to interpret from evolutionary considerations.

The gram-negative bacterium *Pectobacterium* is a pathogen causing soft-rot disease in a range of plant types [10–12]. Strains of *Pectobacterium* across the globe are distinguished geographically, leading to suggestions that they have evolved to specifically rot-endemic plants [13]. Plants have complex, species-specific mechanisms for withholding iron from pathogens such as *Pectobacterium* [14–16], and successful pathogens therefore need to develop species-specific countermeasures [7, 17]. Defying these and other evolutionary relationships, either through agricultural development or inadvertently, plant species can cross continental barriers. By way of example, Australia was isolated from other land masses for approximately 100 million years and is, therefore, highly vulnerable to noxious weeds, given endemic plants have not adapted for competition, and endemic animals might not graze on introduced weed species. Thus, noxious weeds like Angled onion (*Allium triquetrum*) that have been inadvertently introduced into Australia gain a selective advantage in some environments [18]. However, a strain of *Pectobacterium* was recently isolated on these introduced weeds as a potential biological control agent, as it appears inactive against endemic plants yet highly active against Angled onion [19]. This Australian isolate of *Pectobacterium carotovorum* subsp. *cartorvorum* Waldee was shown to produce severe soft-rot symptoms by invading both cortical and vascular tissue of the weed [19].

To characterize this important isolate of *Pectobacterium*, we used whole genome sequencing. Inspired by previous studies on iron acquisition systems required for soft-rot by the phytopathogens, the genome-encoded complement of TBDTs was analyzed, and a *fusA-fusC* operon was identified. The *fusC* gene was found to be closely related to an orphan protein subfamily of plant proteins annotated as M16 proteases, which, in plants, are involved in organelle biology, including subreactions of the protein import pathway. The crystal structure of FusC from *Pectobacterium* demonstrated the structural characteristics of an M16 protease and further revealed a plausible import mechanism for ferredoxin, an iron-bearing protein from plants. We suggest a Brownian ratchet mechanism by which the phytopathogen binds and imports the plant protein in order to scavenge iron and thereby potentiate rot of the plant host.

## Results

### FusC, a plant-type protease found in *Pectobacterium*

**Fig 1:**
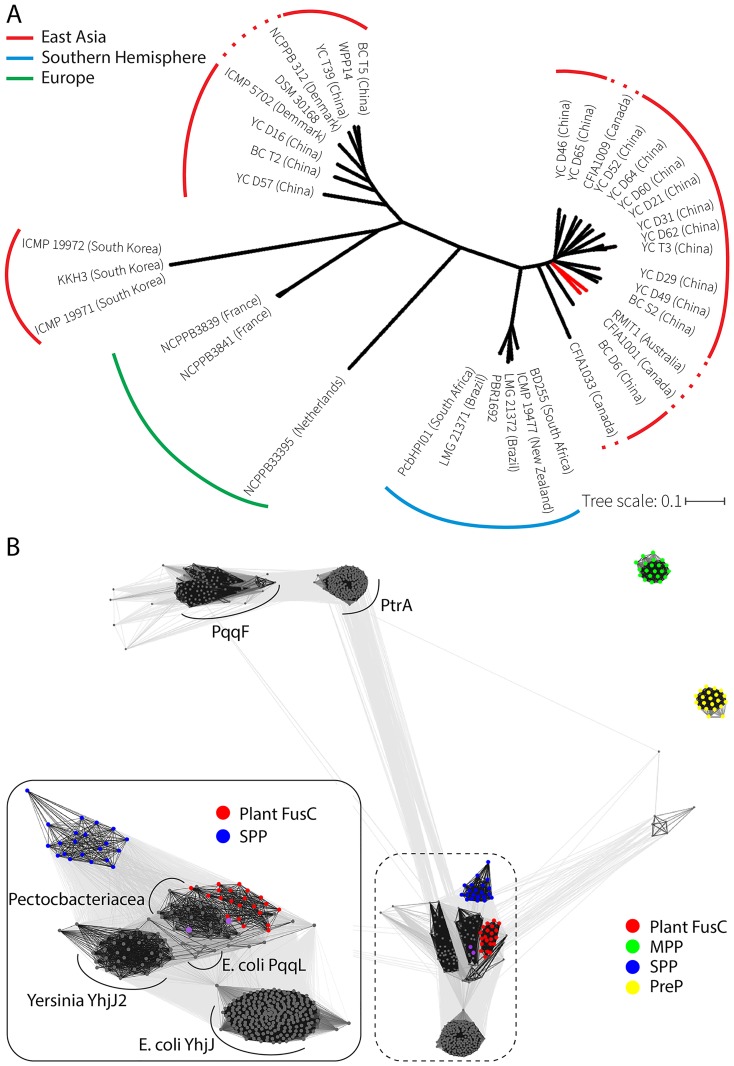
Kinship and iron acquisition in the known species and subspecies of *Pectobacterium*. (A) Molecular phylogeny of *Pectobacterium* species, showing the relationship of the Australian isolate RMIT1 as being *P*. *carotovorum* subsp. *cartorvorum* Waldee. In the analysis shown here, the Australian isolate is closely related to published genomes of *P*. *carotovorum* subsp. *cartorvorum* from China (red branch). Recent whole genome–based phylogenetic analysis suggests that the four recognized subspecies of *P*. *carotovorum* (*actinidiae*, *odoriferum*, *carotovorum*, and *brasiliense*) should be elevated to distinct species [63]. (B) CLANS similarity network analysis depicts homology in protein datasets via all-against-all pairwise BLAST to cluster representations (dots) of individual protein sequences. Gray scale lines are shown between samples, with the most similar sequences shown as black, with an E-value cutoff of 1e−15. The analysis shows that M16 proteases from diverse plants species (S3 Table) cluster into four groups: the MPP group (green), the plastid SPP group (blue), the PreP group (yellow), and a previously unnoticed M16 group we refer to as plant FusC (red). The M16 proteins from Enterobacteriaceae (S1 Data) cluster into five main groups: the PqqF-like sequences, the PtrA-like sequences, the sequences similar to YhjJ from *E*. *coli*, the sequences similar to YhjJ2 from *Yersinia* spp., and a cluster containing FusC proteins mainly from *Pectobacterium* and *Klebsiella* spp. (a minor cluster containing a very conserved set of sequences represented by *E*. *coli* PqqL sits between the FusC, YhjJ2, and YhjJ1 clusters). The CLANS analysis places the bacterial FusC sequence as being most related to the plant FusC sequences. CLANS, cluster analysis of sequences; MPP, mitochondrial processing peptide; PreP, presequence protease; SPP, stromal processing peptidase.

Species of *Pectobacterium* across the globe are distinguished geographically because they have evolved to specifically rot-endemic plants [13]. Phytopathogens can travel with introduced plant species, and whole genome sequencing of the recently identified Australian biocontrol isolate showed genome characteristics of a large group of isolates from China. This is in contradistinction to other *Pectobacterium* isolates from the Southern Hemisphere (“New Zealand,” “South Africa,” “South America”), which form a wholly distinct clade (Fig 1A).

Recently, a ferredoxin receptor, FusA, was identified in the Scottish *P*. *atrosepticum* isolate SCRI1043, encoded in an operon that functions in iron acquisition [9]. To determine whether FusA and other TBDTs for iron acquisition are conserved globally in *Pectobacterium* spp., hidden Markov models (HMMs) were constructed. Genome sequence analysis using the HMM built to detect TBDTs revealed that the Australian isolate RMIT1 encodes 23 TBDTs, many of them typical of iron acquisition systems (Table 1). The most distantly related of these TBDTs shares 82% sequence identity to the ferredoxin receptor FusA from *P*. *atrosepticum* SCRI1043 [9].

**Table 1:** HMM search results on TBDTs in *Pectobacterium*. The HMM score reflects the statistical significance of the hit, the asterisk denotes a lack of statistical significance in the match for the FusA homologue, but structural analysis [9] confirms the FusA sequence as the 23rd example of a TBDT encoded in the genome of *P*. *carotovorum* subsp. *cartorvorum* Waldee RMIT1.

Accession	HMM score	Annotation
MFFDBJGM_00115	483.3	Vitamin B_12_ transporter BtuB
MFFDBJGM_00445	449.4	Hemin receptor
MFFDBJGM_01056	438.6	TonB-dependent heme receptor A
MFFDBJGM_04413	380.1	Ferrienterobactin receptor
MFFDBJGM_03634	365.8	Colicin I receptor
MFFDBJGM_02428	341.4	Colicin I receptor
MFFDBJGM_03797	325.8	Fe^3+^-dicitrate transport protein FecA
MFFDBJGM_02790	315.5	hemoglobin and/or haptoglobin receptor
MFFDBJGM_00472	300.6	Pesticin receptor
MFFDBJGM_00812	290.6	Fe^3+^-pyochelin receptor
MFFDBJGM_03238	282.9	Ferrichrome-iron receptor
MFFDBJGM_01386	267.8	Colicin I receptor
MFFDBJGM_00894	263.0	Ferrichrome-iron receptor
MFFDBJGM_03526	255.3	FhuE receptor
MFFDBJGM_01573	234.7	Ferric aerobactin receptor
MFFDBJGM_02244	228.5	Ferrichrome-iron receptor
MFFDBJGM_03515	182.1	Fe^3+^-dicitrate transport protein FecA
MFFDBJGM_04239	169.2	Vitamin B_12_ transporter BtuB
MFFDBJGM_02272	144.1	Ferric-pseudobactin 358 receptor
MFFDBJGM_00068	137.2	Pesticin receptor
MFFDBJGM_01977	136.9	Ferrichrome receptor FcuA
MFFDBJGM_00214	111.5	Ferrichrome receptor FcuA
MFFDBJGM_04348	41.8*	FusA

Abbreviations: HMM, hidden Markov model; TBDT, TonB-dependent transporter.

Inspection of sequenced *Pectobacterium* genomes revealed that in each case downstream of the *fusA* gene is a gene called *fusC*, previously noted by Walker and colleagues as encoding a protein belonging to the M16 family of proteases [9]. Characteristic features of the M16 metalloprotease family of proteins include being a Zn^2+^-dependent yet ATP-independent protease, having a conserved architecture of two homologous 50-kDa domains that encompass the catalytic chamber, and using electrostatic-mediated interactions to capture substrate proteins [20–22]. The architecture predicted in the conceptual translation of the FusC protein of *Pectobacterium* suggests it would be part of the M16 protease family. In addition to being present in all complete genome sequences from isolates of *Pectobacterium*, the *fusA-fusC* genes are also conserved in two other species of plant pathogen—namely, *Dickeya* and *Klebsiella*. However, assessment of the wealth of complete genome sequence data on isolates of *Klebsiella* spp., which can be commensals and pathogens of plants and can also cause disease in humans [23], showed only a sporadic presence of *fusC* (S1 Fig).

The M16 family of proteases is found broadly across the domains Bacteria and Eukarya. To understand the sequence relationships within this important family of proteases, cluster analysis of sequences (CLANS) was used to classify family members in plants and bacteria. The CLANS analysis highlighted the known mitochondrial and plastid M16 protease subfamilies of mitochondrial processing peptidase (MPP), stromal processing peptidase (SPP), and presequence protease (PreP) (Fig 1B), which function in protein import into these organelles [24]. Interestingly, the analysis revealed that plant genomes also encode a previously undescribed M16 protease subfamily that we refer to as plant FusC (Fig 1B). The nomenclature is appropriate given that the FusC from *Pectobacterium* and *Klebsiella* are most closely related to the novel plant protein (Fig 1B). A number of M16 proteases previously characterized from bacteria, such as PqqL and YhjJ isoforms, are similar to the plant FusC, while the PqqF- and PtrA-type bacterial M16 proteases are more distinct, according to the CLANS data (Fig 1B).

### Crystallization and characterization of FusC from *Pectobacterium*

**Fig 2:**
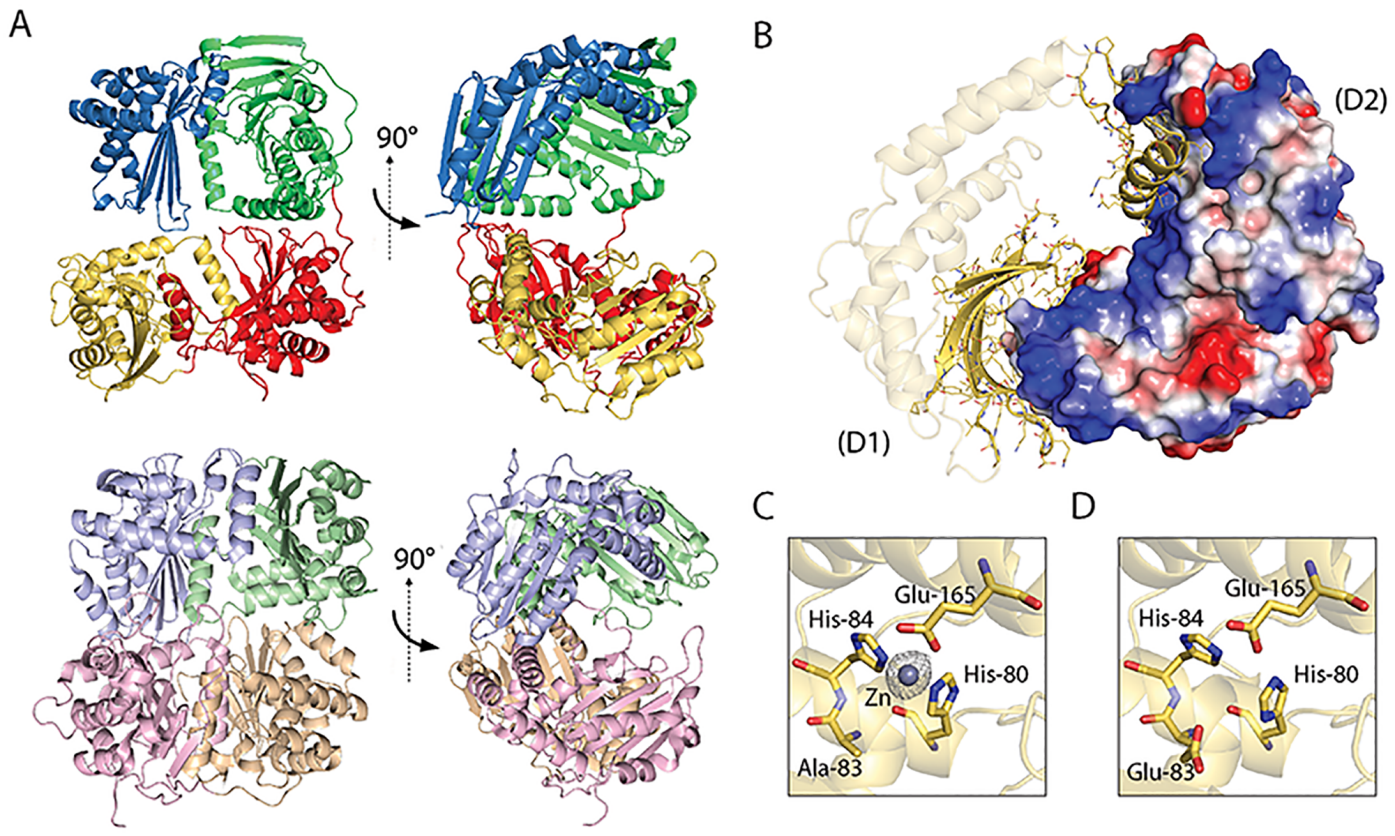
The crystal structure of FusC reveals structural homology to MPP. (A) The crystal structure of FusC (top panel) with the four M16 protease domains colored yellow, red, green, and blue from N- to C-terminus: the halves of FusC form a partially open clamshell structure strikingly similar to the structure of MPP (bottom panel). The MPP (PDB 1HR6) heterodimer has a β-subunit (pink and peach) and α-subunit (pale green and blue). (B) A top-down view of the N-terminal half of FusC showing how domain 1 (labeled “D1”; presented in a cartoon and stick representation) interacts with domain 2 (labeled “D2”; shown with electrostatic surface rendering). (C) The catalytic site of the inactive E^83^A mutant of FusC contains a catalytic zinc ion typical of M16 family proteases. (D) The catalytic site of EDTA-inactivated FusC lacks this metal ion, consistent with its chelation by EDTA. MPP, mitochondrial processing peptide; PDB, Protein Data Bank.

M16 proteases are typically either a dimer of approximately 50-kDa two-domain subunits [25] or an approximately 100-kDa monomer composed of four domains [26]. SignalP [27] predicts that FusC from *Pectobacterium* has a 25-residue N-terminal signal sequence that would direct it into the periplasm and, once processed, would produce a mature protein of approximately 100 kDa. Recombinant FusC was engineered for expression in the periplasm of *Escherichia coli*, and the purified protein was shown to be a monomer in solution (S2 Fig). FusC was crystallized and the structure determined. The structure of the FusC monomer demonstrates conserved features seen in the MPP dimer (Fig 2A). The crystal structure demonstrated that FusC has the four characteristic domains of an M16 protease, creating the entire “clamshell” structure from a single polypeptide (Fig 2A). These four domains of an M16 protease fit together through interactions between the β-sheet and α-helix protruding from the opposing domain, creating half of the clamshell (Fig 2B). The first of these domains (Fig 2C, S3 Fig) would correspond to the catalytically active β-subunit of MPP [28], housing the active site residues with a metal ion coordinated by two histidine residues (H^80^ and H^84^) and a glutamic acid (E^165^). The second domain has an analogous pocket but does not have residues that could coordinate a metal ion for catalysis (S3 Fig). In FusC, domain 3-domain 4 (corresponding to the α-subunit of MPP, which is catalytically inactive [28]) are likewise unable to form an active site (S3 Fig).

#### Cocrystallization reveals binding sites for ferredoxin on FusC

MPP is chemically inactivated in the presence of EDTA, which chelates the crucial zinc cofactor of the metalloprotease [29]. Alternatively, catalytically inactivated versions of MPP can be engineered by mutating a glutamate residue important for catalysis but not for metal binding [30]. The equivalent glutamate residue in FusC, E^83^, was mutated to an alanine residue, and the FusC(E^83^A) mutant was used for structural analysis. The catalytically important metal ion was identified in the FusC(E^83^A) structure (Fig 2C) but was absent in FusC crystallized in the presence of EDTA (Fig 2D).

**Fig 3:**
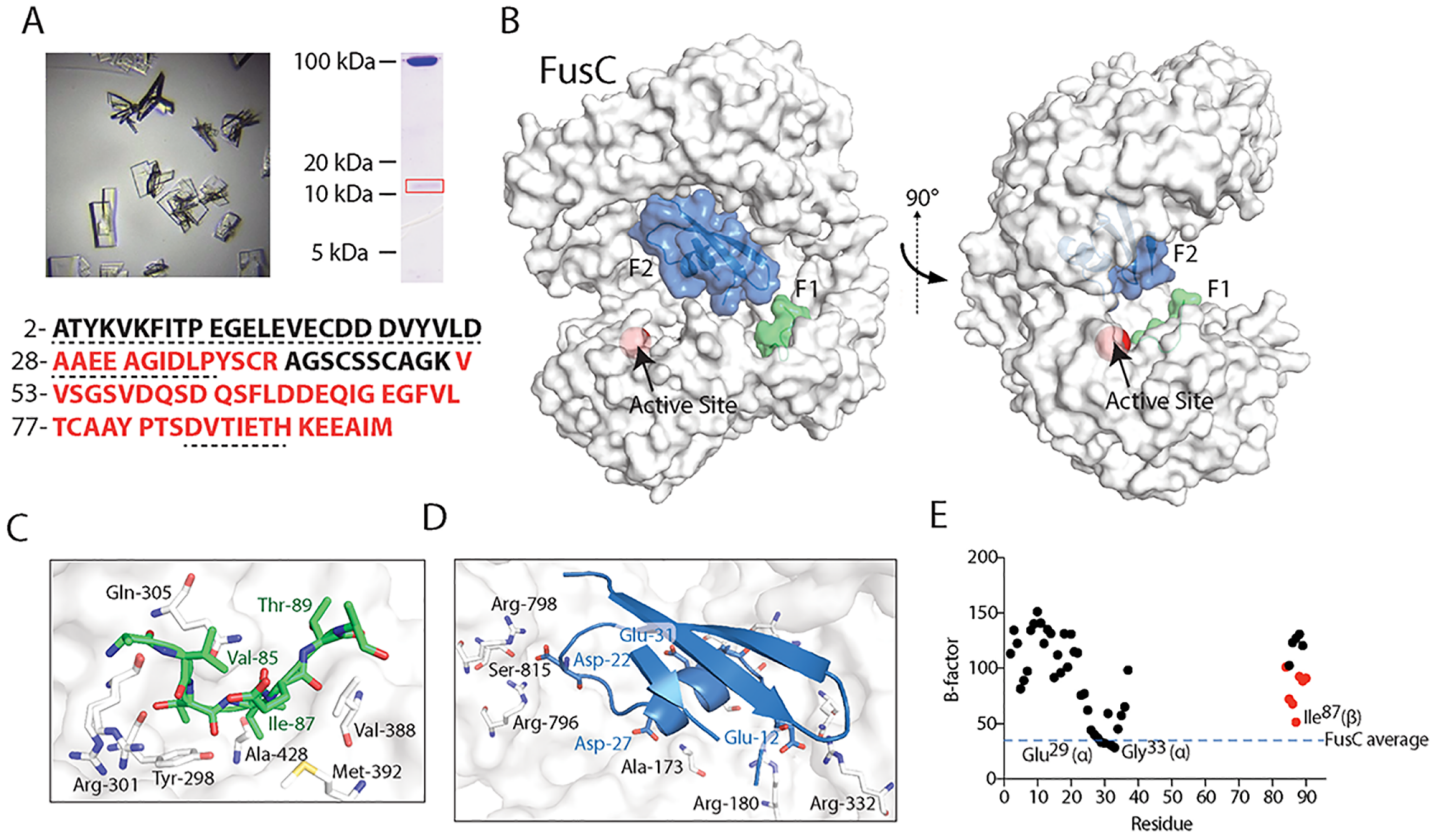
The crystal structure of FusC in complex with ferredoxin reveals two distinct ferredoxin bindings sites. (A) Crystals of the FusC:ferredoxin complex (left) and SDS-PAGE of resolubilized crystals (right) showing that intact ferredoxin (approximately 10 kDa) is present in the crystals and peptides identified by mass spectrometry from regions of a gel excised after SDS-PAGE (red) and modeled in electron density (dotted underline) (below). (B) The crystal structure of FusC in complex with ferredoxin, density corresponding to ferredoxin polypeptide, is present in two distinct binding sites inside the cavity of FusC. F1 corresponds to residues 84–90 of the ferredoxin, while F2 corresponds to residues 2–37 and 86–90. The location of the Zn-containing active site of FusC is shown as a red sphere. (C) The ferredoxin peptide present in the F1 binding site of FusC shown in green as a stick and cartoon representation. FusC is shown in white as a surface and stick representation. V^85^ forms contacts with a hydrophobic pocket in FusC defined by Y^298^, M^392^, and A^428^. (D) The N-terminal portion of ferredoxin present in the F2 binding site of FusC shown in blue as a stick and cartoon representation. FusC is shown as in panel C. Ferredoxin residues D^22^, D^27^, E^12^, and E^31^ form salt bridges with arginine residues at positions 180, 332, 796, and 798 of FusC, stabilizing the ferredoxin–FusC interaction. (E) A plot of temperature factor (B-factor) versus residue number of ferredoxin residues present in the FusC:ferredoxin crystal structure. Red dots correspond to binding site F1 and black dots to binding site F2. Lower B-factors indicate more ordered residues. The average B-factor for residues of FusC is shown as a blue dotted line.

After coincubation with ferredoxin, crystals were formed from FusC(E^83^A) and/or EDTA-treated FusC. SDS-PAGE analysis confirmed that both sets of crystals contained full-length ferredoxin in addition to FusC(E^83^A) or FusC (Fig 3A). The identity of the bound ferredoxin was confirmed by mass spectrometry (Fig 3A). The structure of the FusC:ferredoxin complex was solved using mercury single-wavelength anomalous dispersion (HgSAD) data at 2.3 Å from an ethyl mercury phosphate (C_2_H_5_HgPO_4_)-soaked crystal. The structures of FusC:ferredoxin, FusC:ferredoxin (C_2_H_5_HgPO_4_), and FusC(E^83^A):ferredoxin were refined to 2.7, 2.3, and 1.9 Å, respectively (S1 Table). In these crystal forms, peptide densities were detected that correspond to the plant ferredoxin. These peptides define two distinct binding sites for ferredoxin in the cavity of the FusC structure (Fig 3B, S4 Fig).

Density corresponding to a ferredoxin polypeptide was observed in the crystal structure at a site referred to as F1. This site tethers the ferredoxin by interaction of residues 84–90 into a hydrophobic pocket in FusC (Fig 3C). This pocket is distant from the active site (Fig 3B). At binding site F2, segments of a ferredoxin molecule corresponding to the N-terminal 37 residues and the third β-strand of ferredoxin (residues 86–90) were visualized (Fig 3D). The B-factors for the ferredoxin α-helix bound to this site are comparable to the rest of FusC (Fig 3E), with a number of salt bridges between positively charged residues in FusC and negatively charged residues in ferredoxin contributing to this stability (S4 Fig, S2 Table). SDS-PAGE analysis of the crystals showed that the bound ferredoxin is intact (Fig 3A). This means both (i) that the C-terminal region of a plant ferredoxin can be stably bound by FusC and (ii) that the absence of electron density for the middle part of ferredoxin bound at this site results from disorder in ferredoxin residues 38–85. Further evidence for a partial unfolding of ferredoxin comes from investigation of the constraints from the cavity in which ferredoxin binds. Attempts at docking the folded ferredoxin into the cavity by structural superimposition onto F2 leads to a large number of clashes between the ferredoxin and FusC: 90 contacts <2.3 Å and 22 contacts <1.5 Å are observed (S5 Fig). Taken together with the fact that these clashes occur in the region of the ferredoxin that is not resolved in the crystal structure, we conclude that in order for FusC to bind ferredoxin to form the FusC:ferredoxin complex, ferredoxin must be distorted through partial unfolding (S5 Fig). Furthermore, given that residues 86–90 of ferredoxin are capable of interacting with either F1 or F2, these must represent alternative binding events on two different populations of FusC within the crystal lattice.

### Ferredoxin induces a clamping of FusC in solution

In protein import pathways, the concept of a Brownian ratchet is invoked to explain a means to energize protein movement across a membrane, particularly when some degree of protein unfolding is involved [31]. Such a mechanism requires that a factor on the *trans* side of the membrane can clamp onto a short segment of the substrate protein to transform the nondirectional Brownian motions of the receptor-bound substrate into a vectorial motion across the membrane [32]. Being in the periplasm, FusC would be located on the *trans* side of the bacterial outer membrane and so was tested for the characteristics required of a factor mediating a Brownian ratchet.

**Fig 4:**
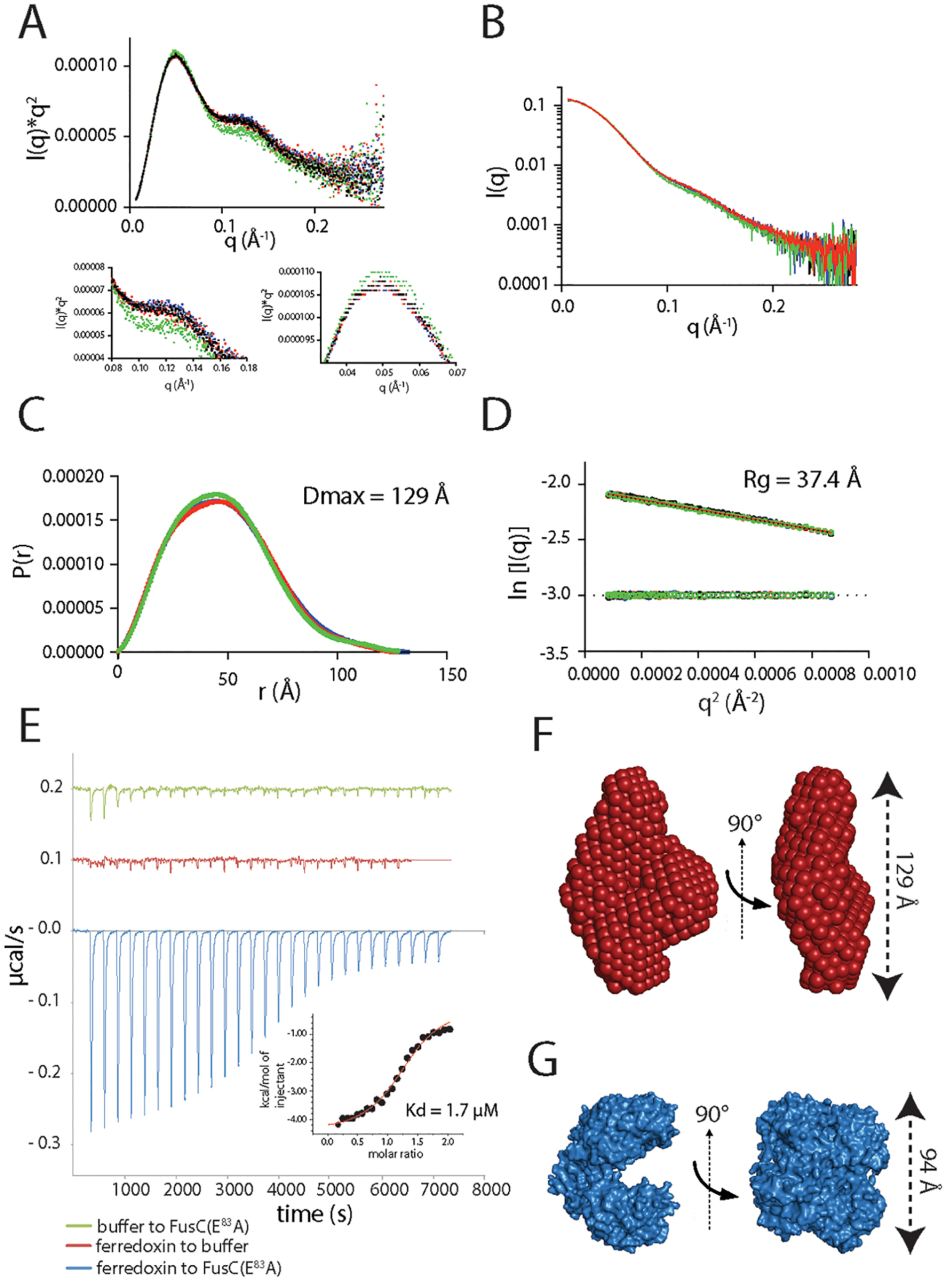
SAXS shows that FusC is flexible in solution and undergoes a conformational change on ferredoxin binding. (A) SEC-SAXS analysis of FusC and FusC(E^83^A) with and without the addition of ferredoxin. FusC is shown in the absence (black) and presence (blue) of ferredoxin; FusC(E^83^A) is shown in its absence (red) and presence (green). The Kratky plot of FusC data suggests a multidomain structure with some interdomain flexibility. Perturbance of FusC(E^83^A) in the presence of ferredoxin is observed. See S2 Data. (B) Scattering angle versus intensity plot for FusC. See S2 Data. (C) P_r_ distribution showing that FusC has a maximum dimension of 129 Å, adopting an elongated conformation in solution. See S2 Data. (D) A plot of the Guinier region of FusC SAXS showing the particle has a radius of gyration of 37.4 Å. See S2 Data. (E) An ITC isotherm of a titration of ferredoxin. Saturatable heats of binding were observed through the titration of ferredoxin into a sample of FusC (blue) but not for a titration of the equivalent buffer into a sample of FusC (green) nor for a titration of ferredoxin into a sample of buffer lacking FusC (red). The fitted binding curve yielded reaction kinetics (Table 2) and a disassociation constant (Kd) of 1.7 μM. See S2 Data. (F) Ab initio model of FusC in solution, generated using DAMMIF, shows that FusC can adopt an elongated confirmation in solution. (G) A surface model of the crystal structure of FusC, which has a maximum dimension of 94 Å. All SAXS data (used to generate plots and models in panels A, B, C, D, and F) have been deposited at the SASBDB, accession: SASDDQ6, SASDDR6, SASDDS6, SASDDT6. ITC, isothermal titration calorimetry; SASBDB, Small Angle Scattering Biological Databank; SAXS, small-angle X-ray scattering; SEC-SAXS, size-exclusion chromatography coupled with small-angle X-ray scattering.

Small-angle X-ray scattering (SAXS) analysis of FusC demonstrated that the protein has interdomain flexibility in solution (Fig 4A). To explore the possibility of FusC providing a Brownian ratchet for ferredoxin import, it was subjected to size-exclusion chromatography coupled with small-angle X-ray scattering (SEC-SAXS). In the absence of ferredoxin, the scattering curves observed for both FusC and FusC(E^83^A) are identical (Fig 4A and 4B). The maximum dimension (D_max_) of Apo-FusC in solution is 129 Å (Fig 4C–4F). This corresponds with the dimensions of two M16 protease clamshells stacked end on end, suggesting FusC can adopt a fully open conformation. This observation contrasts with the partially closed clamshell structure observed in the FusC:ferredoxin crystal structure, which has a D_max_ of 94 Å (Fig 4G). The D_max_ of FusC and its flexibility in solution suggest that its clamshell domains can adopt a range of conformations from fully open to at least partially closed.

**Fig 5:**
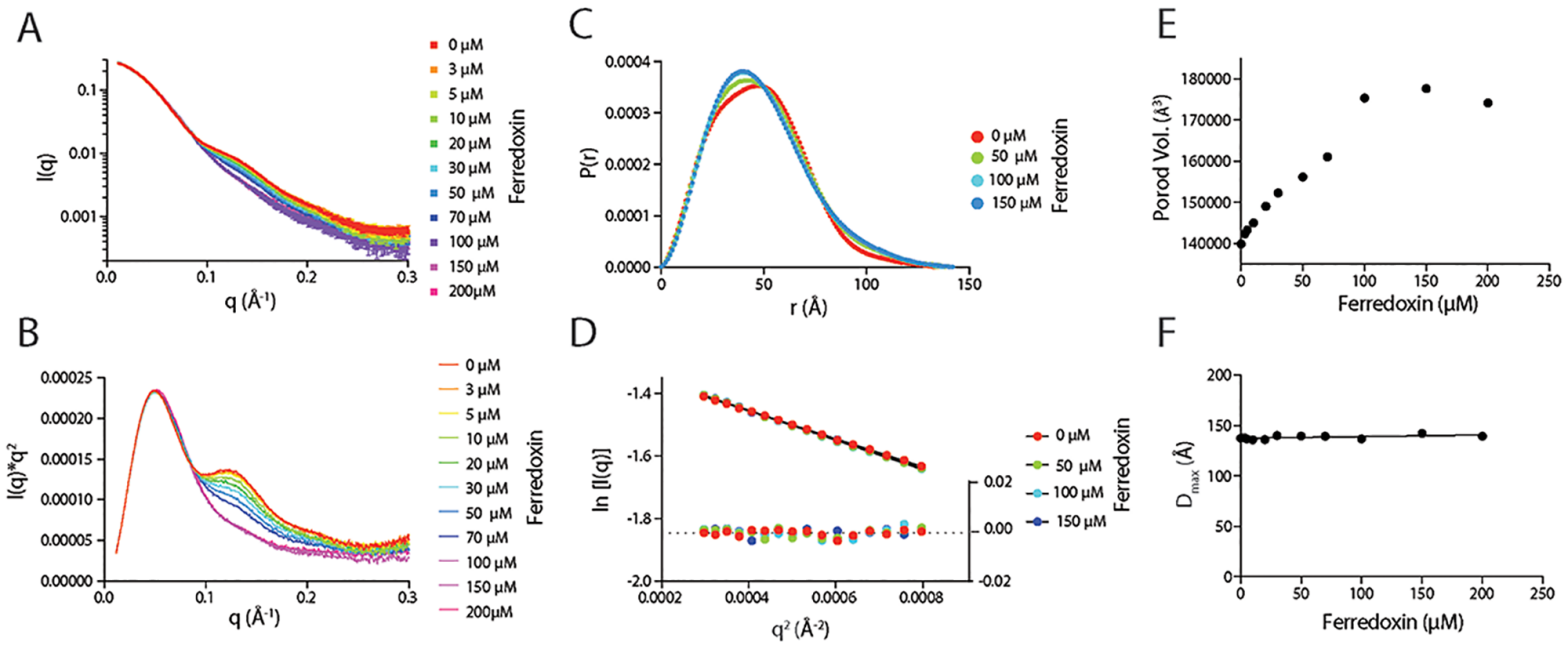
SAXS scattering shows that FusC(E^83^A) undergoes major conformational change upon ferredoxin binding but remains elongated in solution. Static-SAXS titration of ferredoxin into FusC (30 μM) mapping conformational changes that occur in FusC upon ferredoxin binding. (A) Scattering angle versus intensity plot for FusC(E^83^A) with increasing concentration of ferredoxin. (B) Kratky plot of FusC(E^83^A) scattering showing a shift from a multidomain structure to a single domain upon ferredoxin binding. (C) P_r_ distribution of FusC(E^83^A) in the absence of ferredoxin (0 μM), partially saturated (50 μM), and saturated (100 and 150 μM), showing a conformational tightening of FusC upon ferredoxin binding. (D) A plot of the Guinier region of FusC(E^83^A) at increasing ferredoxin concentration. (E) A plot of volume of FusC(E^83^A) with increasing ferredoxin concentration. The observed increase and plateau of Porod volume is indicative of ferredoxin binding and saturation of FusC(E^83^A). (F) A plot of the maximum dimension of FusC(E^83^A) in solution, showing that the protease remains elongated in solution when in complex with ferredoxin. All SAXS data used to generate plots in this figure have been deposited at SASBDB, accession: SASDDU6. SASDDV6, SASDDW6, SASDDX6, SASDDY6, SASDDZ6, SASDD27, SASDD37, SASDD47, SASDD57, SASDD67, SASDD77. SASBDB, Small Angle Scattering Biological Databank; SAXS, small-angle X-ray scattering.

When FusC was mixed with a 3:1 molar excess of ferredoxin prior to SEC-SAXS, no changes were observed in its scattering profile. When the equivalent experiment was run using the FusC(E^83^A) mutant in the presence of ferredoxin, the scattering of FusC(E^83^A) altered, with a decrease in the biomodal nature of the population of FusC(E^83^A) in solution (Fig 4A). The ferredoxin-induced perturbance of the FusC(E^83^A) spectra was small, suggesting that the FusC:ferredoxin complex disassociates over the time course of the size-exclusion chromatography (SEC) experiment. To test this hypothesis, the binding kinetics of FusC(E^83^A) with ferredoxin were determined using isothermal titration calorimetry (ITC) (Table 2). The *K*_d_ of the FusC(E^83^A):ferredoxin complex was found to be 1.9 μM (Fig 4E). Given this affinity and the prospect of dissociation in this range, static-SAXS was utilized to further interrogate FusC:ferredoxin complex formation in solution. Ferredoxin was titrated into FusC(E^83^A) at a range of concentrations from 0:1 to 10:1 molar ratio of ferredoxin to FusC(E^83^A), and a change in scattering of FusC was observed. With increasing ferredoxin concentration, the scattering of FusC(E^83^A) shifted from a biomodal to monomodal distribution that saturated at a 3:1 molar ratio of ferredoxin to FusC (Fig 5A–5F). While the mass of FusC increased with ferredoxin binding, the D_max_ of the particle remained constant, demonstrating that FusC(E^83^A) remains flexible even when bound to ferredoxin. Considering both the observed crystal contacts and the various SAXS analyses, we conclude that FusC can clamp onto ferredoxin and that it does so via a C-terminal segment of its ferredoxin substrate.

**Table 2:** ITC-derived binding parameters for FusC(E^83^A) and ferredoxin.

FusC concentration (μM)	200		
Ferredoxin concentration (μM)	22		
Reaction	1	2	Average
**N**	1.125	1.061	1.09
**Ka (M-1)**	4.52E+05	8.05E+05	6.29E+05
**Kd (M)**	2.21E−06	1.24E−06	1.73E−06
**delH (kcal/mol)**	−5.20	−4.91	−5.06
**delS (kcal/mol/°K)**	0.00822	0.0106	0.01
**temp (°K)**	299.15	299.15	299.15
**delG (kcal/mol)**	−7.7	−8.1	−7.9

Abbreviation: ITC, isothermal titration calorimetry.

## Discussion

Phytopathogens can have myriad strategies to dominate their necrotic environments. Soft rot of host tissues depends on specific protein-secretion systems to deliver proteins with hydrolytic activity into the tissues, and growth and replication of bacteria in this rot are dependent on access to iron from the rotting tissue [33, 34]. Under conditions of iron limitation, growth of a model species of *Pectobacterium* was shown to be supported by plant ferredoxin as an iron source [35]. This has led to the understanding that a pathway for iron acquisition from host proteins is a survival strategy for *Pectobacterium*, and the outer membrane receptor responsible for ferredoxin import into *P*. *atrosepticum* isolate SCRI1043 is the TBDT FusA [9]. How ferredoxin, a tightly folded protein stabilized by its iron cofactor, could be imported across the bacterial outer membrane into the bacterial periplasm had remained a mystery of cell biology.

Given the size of ferredoxin (minimal diameter 35 Å) and the unchangeable internal diameter of the channel in the β-barrel protein FusA (maximal diameter of 34 Å after removal of the plug domain), at least some degree of unfolding of ferredoxin would be required for its import across the *Pectobacterium* outer membrane. How does a bacterium, even a specialist phytopathogen, evolve a means to transport a plant iron–containing protein across its outer membrane? Clues come from the “greasy slide” mechanism by which oligosaccharides can be imported through the maltoporin LamB [36], but, in every case known, translocation of a protein across a membrane requires complex, energy-requiring processes, catalyzed by factors that can recognize the protein substrate of interest. Our analysis suggests that *Pectobacterium* spp. have acquired, either by horizontal gene transfer or by convergent evolution, the FusC subclass of M16 proteases related to the plant FusC proteins found in their hosts.

**Fig 6:**
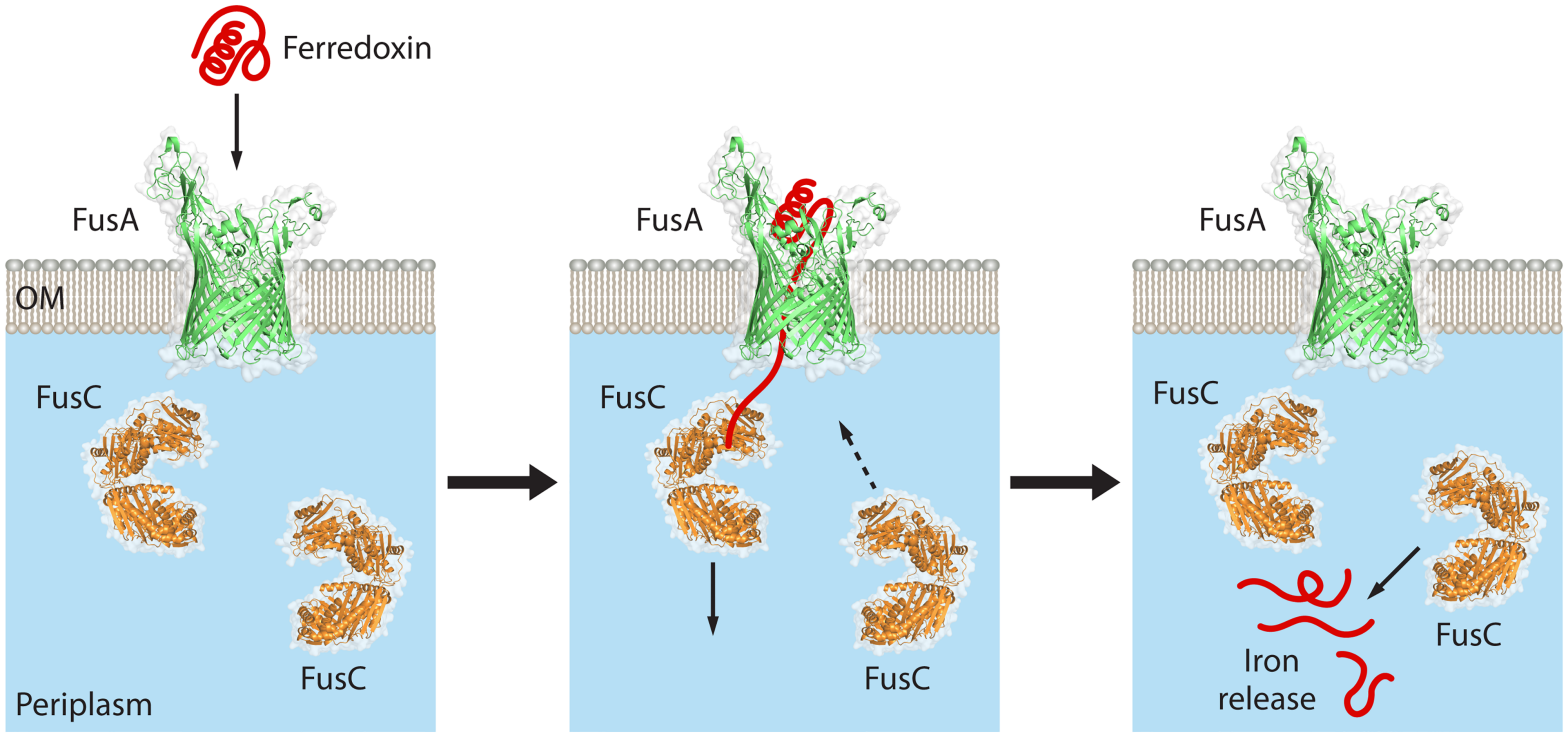
A Brownian ratchet model for ferredoxin import into *Pectobacterium*. The TBDT FusA is a β-barrel protein in the outer membrane (labeled “OM”), with the M16 peptidase FusC on the *trans* side of the outer membrane, in the periplasm. Initial interactions of plant ferredoxin with the *cis* face of FusA have been modeled, and complementary surfaces would provide for a docking reaction [9]. As discussed in the text, some degree of unfolding of ferredoxin is required in order to allow its entry into the translocation channel of the FusA β-barrel. Engagement of an unfolded ferredoxin segment to FusC would establish the conditions for a Brownian ratchet to drive vectorial movement and thereby power protein import through the channel of the FusA β-barrel domain. Given the size of ferredoxin, a complete unfolding of the protein would not be required. Liberation of iron would require proteolysis of ferredoxin by either FusC or another protease in the periplasm. TBDT, TonB-dependent transporter.

In other Brownian ratchet models, protein translocation across a membrane is achieved through Brownian motion “breathing” of a terminal segment of the substrate protein [31, 32]. Such a mechanism could be invoked for the C-terminal segment of ferredoxin, which was observed fixed to the noncatalytic F1 site on FusC. This type of Brownian motion in the C-terminal segment may be enhanced once ferredoxin contacts the “glove” of FusA [9]. By analogy to the process of protein import into mitochondria [31, 32], we suggest a model whereby a noncatalytic ferredoxin-binding site in FusC would assist driving ferredoxin import as a Brownian ratchet (Fig 6). We are unaware that any of the other clades of bacterial M16-peptidases have such a function.

The concept of a Brownian ratchet provides for a net displacement of a segment of polypeptide through a translocation channel by capturing the polypeptide on the *trans* side of the membrane [37]. This capture requires some form of clamp to lock on to the substrate polypeptide, which would otherwise diffuse freely backward and forward through the channel [31, 32, 37]. Even for very large polypeptides, theoretical considerations show that the driving force provided by a Brownian ratchet is more than enough to ensure vectorial translocation across the membrane [37]. In the specific case of a small protein like ferredoxin, with a diameter only slightly greater that the translocation pore itself, unfolding of the C-terminal segment would both decrease the substrate diameter and provide a segment of polypeptide to be captured on the *trans* (periplasmic) side of the channel (Fig 6).

Like the M16 proteases found in bacteria, plants and other eukaryotes also have M16 proteases. Two well-characterized examples, MPP and SPP, work as processing peptidases in protein import pathways. The MPP (formerly MPP/PEP) binds substrate proteins in transit through the translocon of the inner mitochondrial membrane [28, 38, 39]. In plastids, the SPP binds the precursor form of ferredoxin and clips the stromal targeting sequence to generate the mature form of ferredoxin, which can then be folded into the active iron-sulfur protein to participate in photosynthesis [40]. While the function of PreP in degrading peptides, damaged, and nonnative proteins in organelles is well characterized [24], there is as yet no information on the function of the plant FusC that was discovered in our study. It is tempting to speculate that plant FusC might be involved in the turnover of ferredoxin, given the extensive quality control that is required to limit levels of other oxidatively damaged proteins in the process of photosynthesis [41] and the equivalent role of the malarial parasite M16 protease falcilysin that catalyzes turnover of the iron protein hemoglobin [42].

The components that mediate protein import into eukaryotic organelles, such as mitochondria and plastids, evolved from proteins present in their bacterial ancestors [43–45]. We suggest that the import of ferredoxin into *Pectobacterium* makes use of a mechanism analogous to that used in eukaryotic organelles, even using structurally related elements and a concept borrowed from the more complex protein import systems of plastids and mitochondria.

## Materials and methods

### CLANS analysis

Enterobacteriaceae M16 protein sequences (Dataset S1) were obtained from InterPro IPR011765 “Peptidase M16, N-terminal,” and redundancy was minimized using CD-HIT with a 0.98 cutoff [46]. FusC-related plant sequences were initially identified through a BLAST search using *Pectobacterium* FusC as a query sequence. In order to cover a broad phylogenetic distance, plant M16 protein sequences were manually chosen such as to include species of green algae, moss, and monocotyledon and dicotyledon plants (S3 Table). A uniform, representative set of plant species (i.e., the same genome projects) was used for the MPP, SPP, plant FusC, and PreP sequences. The plant sequences were combined with the enterobacterial M16 sequences, and the sequences were classified by an all-against-all BLAST, clustered based on pairwise similarities, and visualized with CLANS with a E-value cut off of 1 × 10^−15^ [47].

While it has not been studied in detail, sequence characteristics of the plant FusC protein from *Arabidopsis thaliana* can be found at the genome project site (https://www.arabidopsis.org/servlets/TairObject?type=locus&name=AT5G56730).

### Comparative genomics

The genome of *P*. *carotovorum* subsp. *cartorvorum* Waldee RMIT1 was sequenced by the Wellcome Trust Sanger Institute genome sequencing facility. Illumina sequencing libraries (Illumina, San Diego, CA, United States of America) were prepared with a 450-bp insert size and were sequenced (Illumina HiSeq2000). The long paired-end reads (100 bp) were assembled [48](https://github.com/sanger-pathogens/) and annotated using prokka [49]. Whole genome sequence data were annotated with prokka (http://www.vicbioinformatics.com/software.prokka.shtml) and yielded the following summary statistics: Contigs: 99—Bases: 4995997—tmRNA: 1—rRNA: 4—CDS: 4461—tRNA: 55—repeat_region: 3. To analyze the phylogenetic relationship of *P*. *carotovorum* subsp. *cartorvorum* Waldee RMIT1 compared with other related strains, a tree was rendered using iTOL (http://itol.embl.de). Whole genome sequence data for the other *Pectobacterium* spp. were accessed (https://www.ncbi.nlm.nih.gov/genome/genomes/1799) via NCBI. Ultimately, a total of 37 sequenced genomes were used for constructing the phylogenetic trees.

To map plant FusC sequences in respect of phylogenetic relationships of globally sampled *Klebsiella* genomes, the genome sequence data and associated metadata—including species, sample source, location, and date of isolation—were obtained from the NCBI Pathogen Detection project database via micro-react.org (https://microreact.org/project/ncbi-klebsiella) and visualized using Figtree v1.4.3 (http://tree.bio.ed.ac.uk/software/figtree/). To identify *Klebsiella* strains carrying peptidase homologues, we used NCBI tblastn with an E-value cutoff of 1e−05 to query a representative M16 peptidase sequence (genbank ID: WP049102367.1) against the NCBI nucleotide collection databases, and the location of the positive BLAST query results were mapped onto the *Klebsiella* phylogeny.

### TBDT HMM search

TBDT sequences were collected from UniProt and literature [50, 51], duplicates were removed, and the remaining 87 samples were aligned with T-Coffee [52] to generate the formatted alignment results as input file for HMM training. Then, we used HMMER [53] to train the HMM and employed this model to search against the *Pectobacterium* genome dataset. Finally, 23 putative sequences with high confidence were retrieved (Table 1). After comparing them with the original dataset, we found no overlap. We added these putative sequences into the original dataset and trained a new HMM to iteratively search against the *Pectobacterium* genome dataset. No further putative sequences were retrieved in this subsequent search.

### Protein expression and purification

A DNA fragment encoding the FusC open reading frame lacking the signal peptide was amplified to contain *Nco*I and *Xho*I restriction sites and cloned into a modified pET20b vector with sequence that would add an N-terminal polyhistidine tag followed by a TEV cleavage site to the encoded recombinant protein. The resulting vector was designated pETFusC. To express a FusC(E^83^A) mutant, whole plasmid mutagenesis was utilized [54]. To amplify pETFusC incorporating a single base pair change from “A” to “C” (at the codon indicated in bold), PCR was used with the overlapping oligonucleotide primers: Forward—GTAGCGCACATGGTC**GCA**CACATGGTTTTTCGTG; Reverse—CACGAAAAACCATGTGTG**CGA**CCATGTGCGCTAC. The product from this reaction was digested with *Dpn*I for 1 h at 37 °C and then transformed into *E*. *coli* Top10 cells. Plasmid DNA was prepared from these colonies and sequenced to confirm the A to C mutation: the resulting plasmid was designated pETFusCE^83^A. Both vectors were transformed into *E*. *coli* BL21 (DE3) C41 for protein expression. Protein expression was performed in terrific broth (12 g tryptone, 24 g yeast extract, 61.3 g K_2_HPO_4_, 11.55 g KH_2_PO_4_, 10 g glycerol) containing 100 mg.ml^−1^ ampicillin for plasmid selection. Bacterial cultures were incubated at 37 °C until OD_600_ of 1.0, then protein expression was induced with 0.3 mM IPTG, and incubation continued for 14 h at 25 °C. Cells were harvested by centrifugation and lysed using a cell disruptor (Emulseflex) in Ni-binding buffer (50 mM Tris, 500 mM NaCl, 20 mM imidazole [pH 7.9]) containing 0.1 mg.ml^−1^ lysozyme, 0.05 mg.ml^−1^ DNase1, and cOmplete Protease Inhibitor Cocktail (Roche). The resulting lysate was clarified by centrifugation and applied to Ni-NTA agarose, followed by washing with 10× column volumes of Ni-binding buffer and elution of protein with a stepwise gradient of Ni-gradient buffer (50 mM Tris, 500 mM NaCl, using steps of 25, 50, 125, and 250 mM Imidazole [pH7.9]). Fractions containing eluted recombinant protein were pooled and applied to a 26/600 Superdex 200 size-exclusion column equilibrated in SEC buffer (50 mM Tris, 200 mM NaCl [pH 7.9]). Fractions from SEC were pooled and incubated with 0.5 mg/ml^−1^ TEV protease and 1 mM DTT at 20 °C for 4 h to cleave the N-terminal 10×His tag. This solution was then passed through a 1-ml Ni-NTA agarose column, with the flow-through containing purified, TEV-cleaved FusC collected. Purified FusC was concentrated to 10 mg/ml and then snap frozen for storage at −80 °C.

As a model ferredoxin, the protein from the plant *A*. *thaliana* was produced using vector previously described [9], with the protein purified using the same protocol as for FusC.

### Protein crystallization, data collection, and structure solution

Purified FusC alone or in combination with ferredoxin (3:1 molar ratio ferredoxin to FusC) was screened for crystallization conditions using commercially available screens (approximately 800 conditions). No crystals were obtained for FusC alone; however, crystals grew in a number of conditions with FusC in combination with ferredoxin. Crystals of FusC only grew in both the presence of both ferredoxin and 3−5 mM EDTA. The presence of FusC and ferredoxin in the crystals was confirmed by collecting crystals with well solution and melting them in H_2_O for analysis by SDS-PAGE. A 10-kDa protein corresponding in size to intact ferredoxin was confirmed to be ferredoxin by mass spectrometry (Fig 4A).

An initial crystallization condition of 0.1 M Bis-Tris propane, 0.2 M NaK phosphate, 20% PEG 3,350 pH 6.5 was chosen for optimization. Crystals were cryoprotected by soaking for 10 min in crystallization solution plus 20% glycerol with or without a small quantity of powdered ethylmercury phosphate and flash cooled in liquid N_2_. Native data were collected at 100 °K at the Australian synchrotron and processed in the space group P22_1_2_1_ to 2.7 Å. Single-wavelength anomalous dispersion (SAD) data from ethylmercury phosphate–soaked crystals were collected using the same parameters as native data, yielding the same space group with data processed to 2.3 Å. The catalytically inactive FusC(E^83^A) mutant was crystallized in the presence of ferredoxin as with wild-type FusC, however, without the 3−5 mM EDTA required for crystallization of wild-type FusC. Crystals grew in 0.2 M NaK phosphate, 20% PEG3350. Crystals were flash cooled and data collected as per wild-type FusC with diffraction data processed to 1.9 Å with the same space group and unit cell parameters as wild-type protein.

Phenix autosol was used to locate heavy atom sites in HgSAD data to perform phasing and density modification [55]. The best phasing solution consisted of 13 Hg sites, most of which had very low occupancy (<0.2), with 4 higher occupancy sites between 0.35 and 0.45. This partial derivatization provided poor initial phases; however, density modification yielded interpretable maps. The FusC:ferredoxin complex structure was built using programs from the Phenix package in addition to manual building using Coot [55, 56]. This FusC model was then used for molecular replacement, using Phaser, of both FusC and FusC(E^83^A) datasets [57]. These models were then built and refined [58].

### ITC

Purified concentrated FusC(E^83^A) and ferredoxin were dialyzed extensively against the same reservoir of ITC buffer (20 mM HEPES, 50 mM NaCl [pH 7.5]). The concentration of proteins post dialysis was determined using absorbance at 280 nM and BCA assay. Proteins were then diluted in ITC buffer to reaction concentrations of 20 μM for FusC(E^83^A) and 200 μM ferredoxin. Protein concentrations were estimated again, following dilution. All ITC experiments were performed using a MicroCal VP-ITC calorimeter. Ten μl ferredoxin was sequentially titrated into the ITC reaction chamber containing FusC(E^83^A). Negative heats of binding were observed, and 27 titrations were performed per reaction, allowing the ferredoxin concentration in the chamber to reach saturation. Control reactions were performed, with titration of either buffer into FusC(E^83^A) or ferredoxin into buffer. These reactions yielded no significant heats of dilution. All reactions were repeated 3 times; binding parameters were calculated from the average of the two best reactions. Representative heats of binding from a single reaction are presented.

### SAXS

SEC-SAXS was performed using Coflow apparatus at the Australian Synchrotron [59, 60]. Purified FusC or FusC(E^83^A) +/− ferredoxin were analyzed at a preinjection concentration of 100 μM for FusC and 300 μM ferredoxin. Chromatography for SEC-SAXS was performed at 22 °C with a 10/30 Superdex S200 column, at a flow rate of 0.4 ml/min in 50 mM Tris, 100 mM NaCl, 5% glycerol, and 0.2% sodium azide [pH7.9]. The inclusion of glycerol and azide were essential to prevent capillary fouling due to photo-oxidation of buffer components. Scattering data were collected for 1-s exposures over a *q* range of 0.0 to 0.3 Å^−1^. A buffer blank for each SEC-SAXS run was prepared by averaging 10–20 frames pre- or postprotein elution. Scattering curves from peaks corresponding to FusC or FusC(E^83^A) were then buffer subtracted, scaled across the elution peak, and compared for interparticle effects. Identical curves (5–10) from each elution were then averaged for analysis. Data were analyzed using the ATSAS package, Scatter, and SOMO solution modeler [61].

For static-SAXS—titrations of ferredoxin into FusC(E^83^A)—the purified proteins were buffer matched in 50 mM Tris, 100 mM NaCl, 5% glycerol [pH 7.9], and a series of samples were prepared with FusC at a constant concentration of 30 μM, and ferredoxin at a range of concentrations 0–300 μM. A series of blanks was also prepared with ferredoxin in buffer at a range of concentrations 0–300 μM. Scattering for samples was collected at 22 °C over a *q* range of 0.0 to 0.4 Å^−1^, with exposures of 1 s, and scattering from the blank of the corresponding ferredoxin concentration was subtracted. Data were analyzed as for SEC-SAXS experiments.

### Analytical ultracentrifugation analysis

Sedimentation velocity (SV) was carried out in a Beckman Coulter Optima analytical ultracentrifuge using an An-50 Ti 8-hole rotor. FusC (370 μl) at concentrations ranging from 0.25 to 2 mg.ml^−1^ was loaded into a 12-mm path-length centerpiece and centrifuged at 40,000 rpm for approximately 6 h at 20 °C. Scans were collected every 20 s using absorbance optics (at 230, 240, and 280 nm; a radial range of 5.8–7.2 cm; and radial step size of 0.005 cm), using a buffer containing 50 mM Tris, 200 mM NaCl, pH 7.9. Data were analyzed with SEDFIT, using the continuous c(s) distribution model [62]. SEDNTERP was used to calculate the partial specific volume, the buffer density, and viscosity at 15 °C and 20 °C.

## Supporting information

S1 Fig Disparate acquisition of *fusC* genes in *Klebsiella* spp. *Klebsiella* genome sequences were visualized using Figtree v1.4.3 (http://tree.bio.ed.ac.uk/software/figtree/). Each terminal node, representing a genome, was colored according to the geographical location for the isolate, based on metadata obtained from the NCBI Pathogen Detection project database via micro-react.org (https://microreact.org/project/ncbi-klebsiella). The two genomes that carry the gene encoding FusC are highlighted in yellow. *Klebsiella pneumoniae* subsp. *rhinoscleromatis* SBS3432 was isolated from an 11-year-old patient in France during 2004, and the FusC sequence showed 82% similarity to the *Pectobacterium* FusC (E-value = 1e−179). *K*. *pneumoniae* subsp. *pneumoniae* Kp52.145 is from an unknown source, and the FusC sequence showed 73% similarity to the *Pectobacterium* FusC (E-value = 4e−79). NCBI, National Center for Biotechnology Information.
(PDF)

S2 Fig Biophysical characterization of purified FusC. Analytical-SEC (upper panel) and analytical ultracentrifugation (lower panel) showing that purified FusC is a monomer of approximately 104 kDa. SEC, size-exclusion chromatography.
(PDF)

S3 Fig Structural comparisons of the catalytic lobes of MPP and FusC. The β-subunit of MPP houses the active site and is represented here in silver coloring. The side-chains of catalytically important residues are shown as sticks, with the metal (Zn2+) cofactor designated as a gray sphere. The equivalent domain of FusC is represented in yellow, and its structural similarity is evidenced by an RMSD between the catalytic domains of MPP and FusC of 1.7 Å. The other domains of FusC are structurally related but quite distinguishable from the catalytic domain: the RMSDs between the catalytic domain of FusC and the other three domains are 6.2 Å (red), 3.4 Å (green), and 5.9 Å (blue). The color-coding of the domains is consistent with that shown in Fig 2A (reproduced here in the inset). MPP, mitochondrial processing peptide; RMSD, root-mean-square deviation.
(PDF)

S4 Fig The FusC:ferredoxin crystal structure electron density. Stereo electron density and model of the refined FusC:ferredoxin crystal structures. Density was prepared through generation of a composite omit map and is contoured to 1.0 σ.
(PDF)

S5 Fig Cocrystallization and structural modeling are consistent in suggesting a partial unfolding of ferredoxin in the FusC:ferredoxin complex. (A) The crystal structure of full-length *Arabidopsis* ferredoxin (4ZHO), docked into the FusC cavity by superimposition with the ferredoxin fragment located at binding site F2. This docking results in a large number of clashes between the ferredoxin and FusC: 90 contacts <2.3 Å and 22 contacts <1.5 Å are observed. FusC is shown as a white/silver surface, and ferredoxin is shown as a blue cartoon model, with the Fe-S cluster as spheres. (B) *Arabidopsis* ferredoxin docked to FusC as in panel A with the ferredoxin shown as a blue surface representation. (C) The docked structure from A ignoring the clashes that would require ferredoxin to be partially unfolded. Green shading shows (i) the structure of the C-terminal segment of ferredoxin as seen in the crystals, bound in site F1, and (ii) where that region of ferredoxin would sit if the bound ferredoxin were folded. The displacement required is at least 21 Å.
(PDF)

S1 Table FusC:ferredoxin crystallographic data collection and refinement statistics. (PDF)

S2 Table FusC:ferredoxin interface statistics. (PDF)

S3 Table Sequence accession data for plants M16 sequences used in the CLANS analysis. CLANS, cluster analysis of sequences.
(PDF)

S1 Data Data underlying this paper. (XLSX)

S2 Data Data underlying this paper. (XLSX)

## Abbreviations

CLANS: cluster analysis of sequences
HgSAD: mercury single-wavelength anomalous dispersion
HMM: hidden Markov model
ITC: isothermal titration calorimetry
MPP: mitochondrial processing peptidase
PDB: Protein Data Bank
PreP: presequence protease
SAD: single-wavelength anomalous dispersion
SASBDB: Small Angle Scattering Biological Databank
SAXS: small-angle X-ray scattering
SEC: size-exclusion chromatography
SEC-SAXS: size-exclusion chromatography coupled with small-angle X-ray scattering
SPP: stromal processing peptidase
SV: sedimentation velocity
TBDT: TonB-dependent transporter
