# Supplementary figures and images for: FusC, a member of the M16 protease family acquired by bacteria for iron piracy against plants
Source: PLoS Biol. 2018 Aug 2;16(8):e2006026. doi: 10.1371/journal.pbio.2006026 (PMC6071955; doi:10.1371/journal.pbio.2006026)

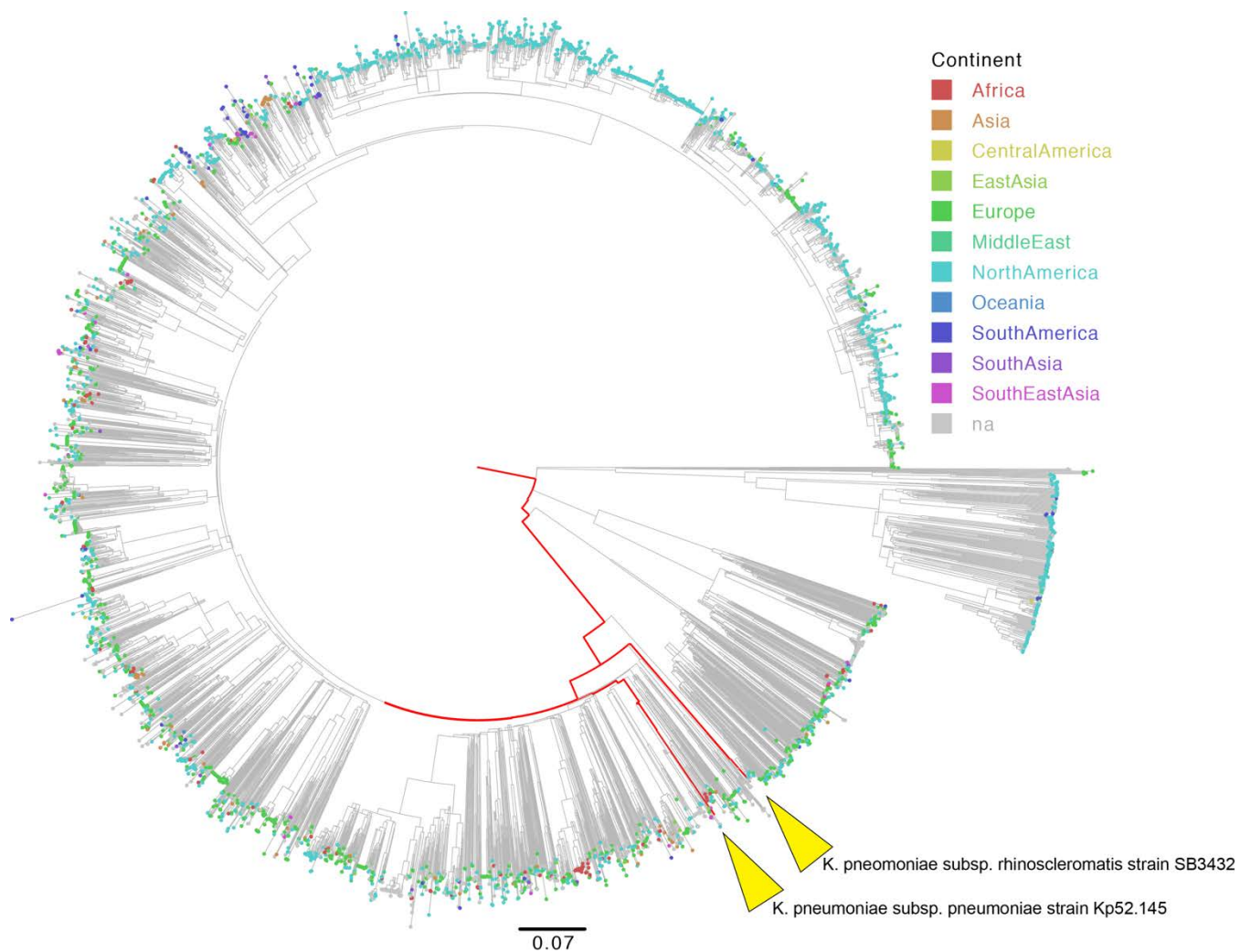

Supplement: S1 Fig — Klebsiella genome sequences were visualized using Figtree v1.4.3 (http://tree.bio.ed.ac.uk/software/figtree/). Each terminal node, representing a genome, was colored according to the geographical location for the isolate, based on metadata obtained from the NCBI Pathogen Detection project database via micro-react.org (https://microreact.org/project/ncbi-klebsiella). The two genomes that carry the gene encoding FusC are highlighted in yellow. Klebsiella pneumoniae subsp. rhinoscleromatis SBS3432 was isolated from an 11-year-old patient in France during 2004, and the FusC sequence showed 82% similarity to the Pectobacterium FusC (E-value = 1e−179). K. pneumoniae subsp. pneumoniae Kp52.145 is from an unknown source, and the FusC sequence showed 73% similarity to the Pectobacterium FusC (E-value = 4e−79). NCBI, National Center for Biotechnology Information. (PDF) [file pbio.2006026.s001.pdf]

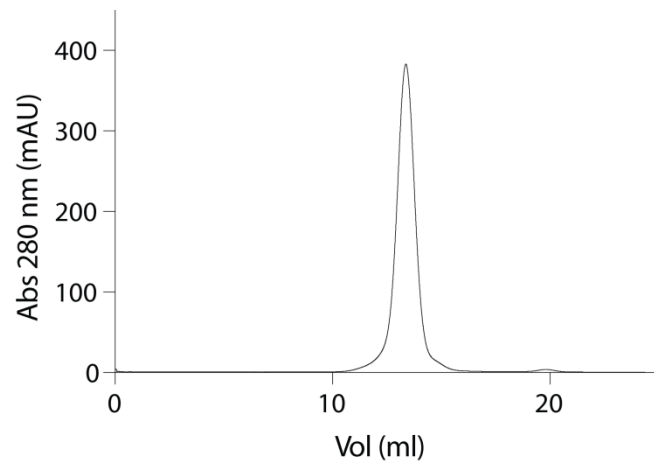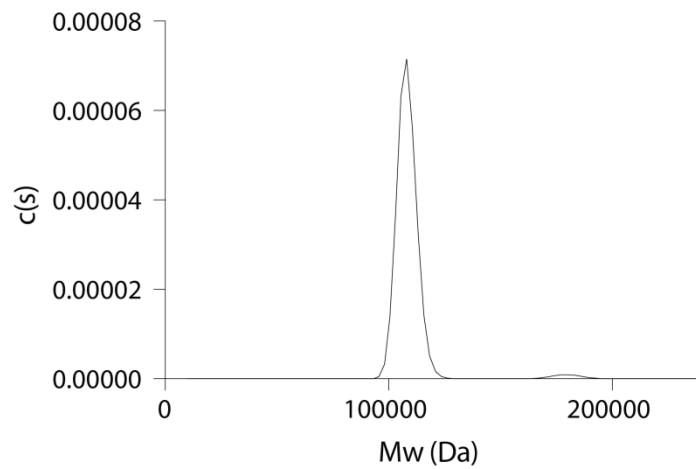

Supplement: S2 Fig — Analytical-SEC (upper panel) and analytical ultracentrifugation (lower panel) showing that purified FusC is a monomer of approximately 104 kDa. SEC, size-exclusion chromatography. (PDF) [file pbio.2006026.s002.pdf]

MPP  $\beta$ -subunit domain 1

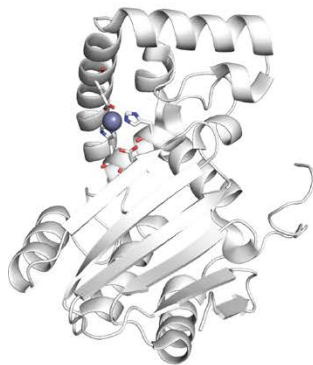

FusC domain 1

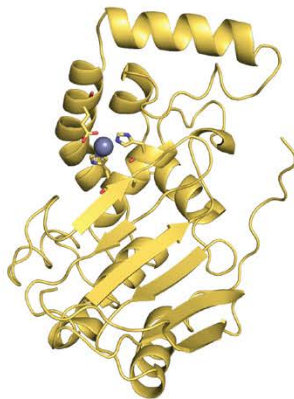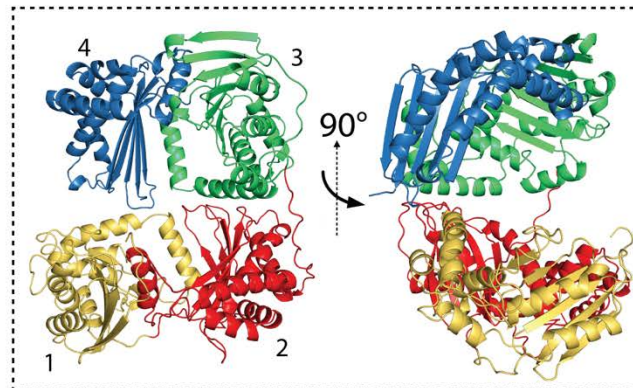

FusC domain 2

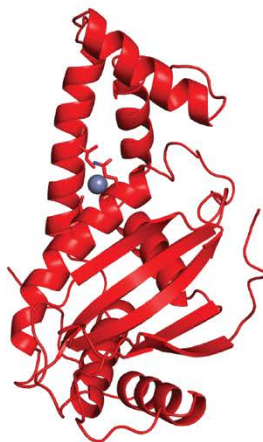

FusC domain 3

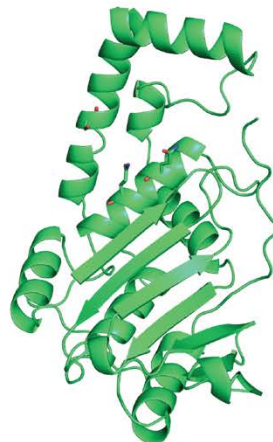

FusC domain 4

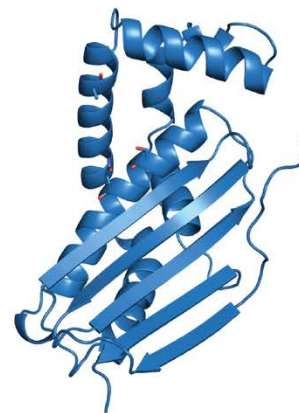

Supplement: S3 Fig — The β-subunit of MPP houses the active site and is represented here in silver coloring. The side-chains of catalytically important residues are shown as sticks, with the metal (Zn2+) cofactor designated as a gray sphere. The equivalent domain of FusC is represented in yellow, and its structural similarity is evidenced by an RMSD between the catalytic domains of MPP and FusC of 1.7 Å. The other domains of FusC are structurally related but quite distinguishable from the catalytic domain: the RMSDs between the catalytic domain of FusC and the other three domains are 6.2 Å (red), 3.4 Å (green), and 5.9 Å (blue). The color-coding of the domains is consistent with that shown in Fig 2A (reproduced here in the inset). MPP, mitochondrial processing peptide; RMSD, root-mean-square deviation. (PDF) [file pbio.2006026.s003.pdf]

## FusC

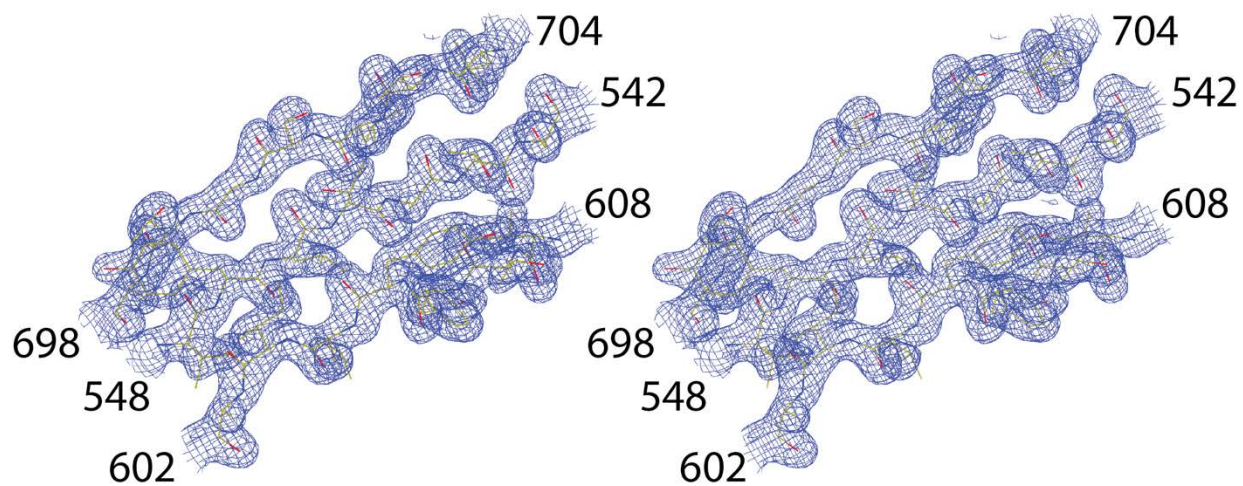

## Ferredoxin Fragment 1

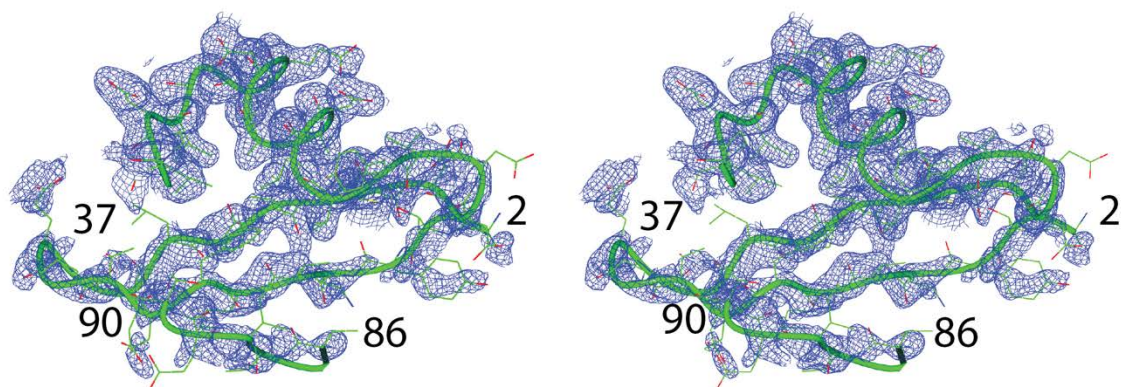

## Ferredoxin Fragment 2

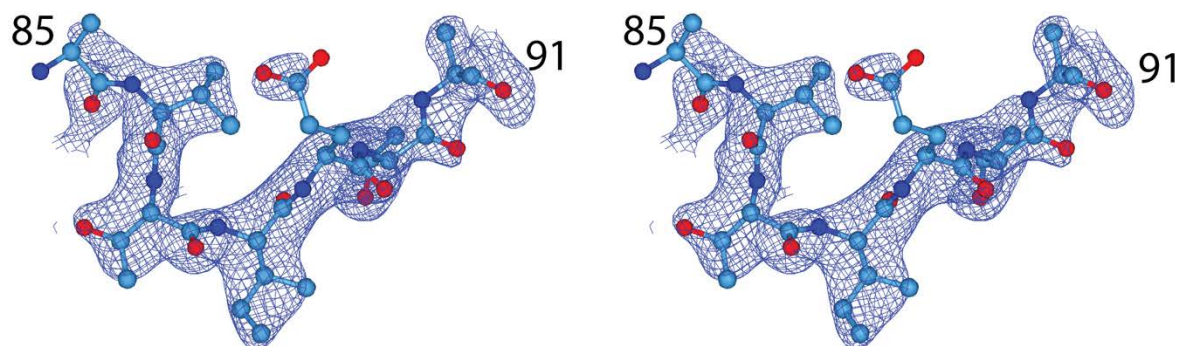

Supplement: S4 Fig — Stereo electron density and model of the refined FusC:ferredoxin crystal structures. Density was prepared through generation of a composite omit map and is contoured to 1.0 σ. (PDF) [file pbio.2006026.s004.pdf]

A

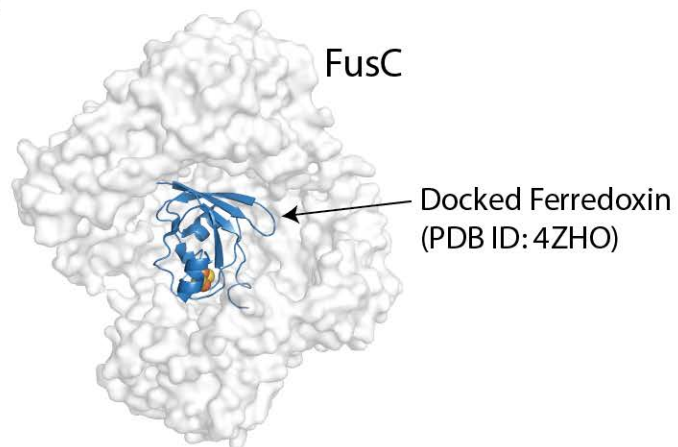

B

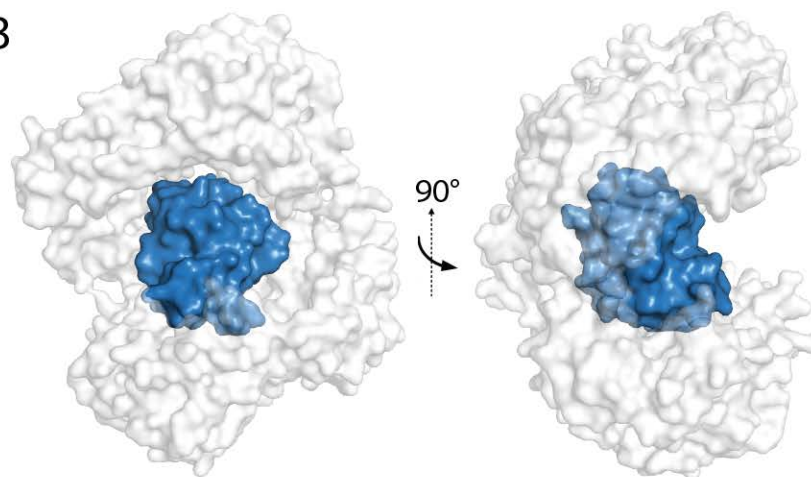

C

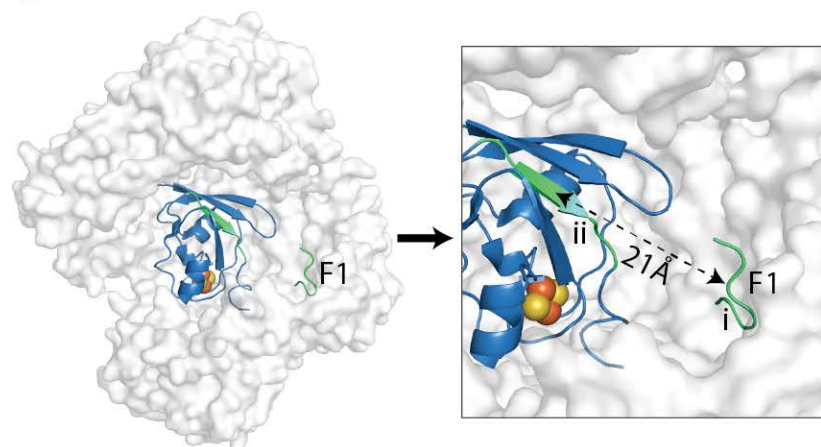

Supplement: S5 Fig — (A) The crystal structure of full-length Arabidopsis ferredoxin (4ZHO), docked into the FusC cavity by superimposition with the ferredoxin fragment located at binding site F2. This docking results in a large number of clashes between the ferredoxin and FusC: 90 contacts <2.3 Å and 22 contacts <1.5 Å are observed. FusC is shown as a white/silver surface, and ferredoxin is shown as a blue cartoon model, with the Fe-S cluster as spheres. (B) Arabidopsis ferredoxin docked to FusC as in panel A with the ferredoxin shown as a blue surface representation. (C) The docked structure from A ignoring the clashes that would require ferredoxin to be partially unfolded. Green shading shows (i) the structure of the C-terminal segment of ferredoxin as seen in the crystals, bound in site F1, and (ii) where that region of ferredoxin would sit if the bound ferredoxin were folded. The displacement required is at least 21 Å. (PDF) [file pbio.2006026.s005.pdf]
