# Supplementary material for: FusC, a member of the M16 protease family acquired by bacteria for iron piracy against plants
Source: PLoS Biol. 2018 Aug 2;16(8):e2006026. doi: 10.1371/journal.pbio.2006026 (PMC6071955; doi:10.1371/journal.pbio.2006026)
Supplement: S1 Table — (PDF) [file pbio.2006026.s006.pdf]

S1 Table. FusC-Ferredoxin crystallographic data collection and refinement statistics.

	FusC-Ferredoxin Hg-SAD	FusC-Ferredoxin Native	FusC-Ferredoxin EtoA
**Data Collection**[a]			
Space Group	$P22_12_1$	$P22_12_1$	$P22_12_1$
Cell Dimensions			
a, b, c (Å)	81.53, 127.37, 132.2	82.58, 127.26, 133.21	81.34, 126.51, 133.54
α, β, γ (°)	90, 90, 90	90, 90, 90	90, 90, 90
Wavelength	0.954	0.954	0.954
Resolution (Å)	47.62-2.30 (2.36-2.30)	48.01-2.70 (2.81-2.70)	47.49-1.90 (1.93-1.90)
R_{merge}	26.0 (301.4)	14.9 (136.3)	11.0 (163.7)
R_{pim}	4.9 (56.5)	7.0 (61.4)	3.2 (69.2)
$I/\sigma(I)$	15.1 (1.5)	7.2 (1.2)	20.1 (1.7)
$CC_{1/2}$	0.998 (0.595)	0.995 (0.538)	0.995 (0.690)
Completeness (%)	100.0 (100.0)	100.0 (100.0)	99.8 (98.9)
Redundancy	29.7 (30.1)	6.6 (6.9)	12.6 (9.4)
**Refinement statistics**			
Resolution (Å)	41.65-2.30	41.29-2.70 (2.81-2.70)	47.49-1.90 (1.93-1.90)
No. relections	61936	39245	107944
R_{work}/R_{free}	18.9/24.2	19.6/24.3	19.7/23.9
No. atoms			
Protein	7581	7576	7581
Ligand / ions	24	0	1
R.m.s deviations			
Bond lengths (Å)	0.009	0.009	0.007
Bond angles (°)	0.928	1.12	0.882

[a] Values in parentheses are for highest-resolution shell.

Data from one crystal was collected for each structure
