# Supplementary material for: FusC, a member of the M16 protease family acquired by bacteria for iron piracy against plants
Source: PLoS Biol. 2018 Aug 2;16(8):e2006026. doi: 10.1371/journal.pbio.2006026 (PMC6071955; doi:10.1371/journal.pbio.2006026)
Supplement: S2 Table — (PDF) [file pbio.2006026.s007.pdf]

S2 Table. FusC-Ferredoxin interface statistics.

HYDROGEN BONDS

	FusC			Ferredoxin Fragment 1		
length (Å)	*number*	*residue*	*atom*	*number*	*residue*	*atom*
3.0	815	Ser	OG	22	ASP	OD2
3.4	172	Val	N	27	ASP	OD1
3.0	173	Ala	N	27	ASP	OD1
3.1	277	Gly	N	30	Glu	O
3.6	276	Ser	OG	30	Glu	O
2.6	276	Ser	OG	30	Glu	OE1
3.5	647	Asn	ND2	31	Glu	O
2.8	643	Arg	NE	32	Ala	O
3.0	280	Gln	NE2	35	Asp	OD2

	FusC			Ferredoxin Fragment 2		
length (Å)	*number*	*residue*	*atom*	*number*	*residue*	*atom*
2.6	305	Gln	OE1	86	Thr	N
3	305	Gln	OE1	87	Ile	N
2.9	305	Gln	NE2	87	Ile	O

SALT BRIDGES

	FusC			Ferredoxin Fragment 1		
length (Å)	*number*	*residue*	*atom*	*number*	*residue*	*atom*
3.4	332	Arg	NE	12	Glu	OE1
3.7	332	Arg	NE	12	Glu	OE2
3.9	332	Arg	NH2	12	Glu	OE2
3.6	798	Arg	NE	22	Asp	OD1
2.7	798	Arg	NH2	22	Asp	OD1
3.2	798	Arg	NH2	22	Asp	OD2
2.9	796	Arg	NH1	22	Asp	OD2
3.8	796	Arg	NH2	22	Asp	OD2

3.6	180	Arg	NH2	30	Glu	OE1
3.5	180	Arg	NH1	30	Glu	OE2
3.0	180	Arg	NH2	30	Glu	OE2
3.1	332	Arg	NH1	35	Asp	OD2
3.5	332	Arg	NH2	35	Asp	OD2
3.6	180	Arg	NE	35	Asp	OD1
3.7	180	Arg	NH2	35	Asp	OD1
