# Supplementary material for: FusC, a member of the M16 protease family acquired by bacteria for iron piracy against plants
Source: PLoS Biol. 2018 Aug 2;16(8):e2006026. doi: 10.1371/journal.pbio.2006026 (PMC6071955; doi:10.1371/journal.pbio.2006026)
Supplement: S3 Table — CLANS, cluster analysis of sequences. (PDF) [file pbio.2006026.s008.pdf]

S3 Table. Sequence accession data for plants M16 sequences used in the CLANS analysis.

Species	MPP	SPP	PreP	plant FusC
Arabidopsis thaliana	NP_566548.1	NP_199054.1	NP_188548.2	NP_001318814
Ostreococcus tauri	XP_003084396.2	XP_003080994.2	OUS48339.1	XP_022839050.1
Micromonas commoda	XP_002508413.1	XP_002506258.1	XP_003061960.1	XP_002502523.1
Bathycoccus prasinos	XP_007509007.1	XP_007510509.1	XP_007514955.1	XP_007513960.1
Psycomitrella patens	XP_001765307.1	XP_001778648.1	XP_001764982.1	XP_001774258.1
Marchantia polymorpha	OAE24872.1	OAE33970.1	OAE21846.1	OAE35728.1
Camelina sativa	XP_010504362.1	XP_010494175.1	XP_010507479.1	XP_010451407.1
Capsella rubella	XP_006299456.1	XP_006279950.1	XP_006296881.1	XP_006279942.1
Ananas comosus	XP_020105364.1	XP_020109242.1	XP_020090952.1	XP_020105782.1
Vitis vinifera	XP_002283426.1	XP_002277544.3	XP_002282024.1	XP_010656234.1
Theobroma cacao	EOY26961.1	EOY15839.1	XP_017971499.1	EOY30542.1
Eucalyptus grandis	XP_010062551.1	XP_010064966.1	XP_010066034.2	XP_010049573.1
Gossypium arboreum	XP_017626663.1	XP_017641919.1	XP_017615507.1	XP_017626046.1
Prunus persica	XP_007215264.1	XP_020423561.1	XP_007200813.2	XP_007204667.1
Sesamum indicum	XP_011093661.1	XP_020552679.1	XP_011088279.1	XP_011093677.1
Beta vulgaris	XP_010688376.1	XP_010679308.1	XP_010691033.1	XP_010667309.1
Lupinus angustifolius	XP_019430315.1	XP_019421373.1	XP_019423118.1	XP_019421253.1
Solanum lycopersicum	XP_004251648.1	XP_010318661.1	XP_004230817.1	XP_004251655.1
Glycine max	XP_003530042.1	XP_006573851.1	XP_003517606.1	XP_006584795.2
Ziziphus jujuba	XP_015902065.1	XP_015880002.1	XP_015892897.1	XP_015889206.1
Selaginella moellendorffii	XP_002987658.1	XP_002986841.1	XP_002979622.1	XP_002985407.1
